# Nanopore Data-Driven T2T Genome Assemblies of *Colletotrichum lini* Strains

**DOI:** 10.3390/jof10120874

**Published:** 2024-12-16

**Authors:** Elizaveta A. Sigova, Ekaterina M. Dvorianinova, Alexander A. Arkhipov, Tatiana A. Rozhmina, Ludmila P. Kudryavtseva, Antoniy M. Kaplun, Yakov V. Bodrov, Valeria A. Pavlova, Elena V. Borkhert, Daiana A. Zhernova, Elena N. Pushkova, Nataliya V. Melnikova, Alexey A. Dmitriev

**Affiliations:** 1 Engelhardt Institute of Molecular Biology, Russian Academy of Sciences, Moscow 119991, Russia; dvorianinova.em@phystech.edu (E.M.D.); arkhipov.aleksandr2.0@gmail.com (A.A.A.); kaplun.am@phystech.edu (A.M.K.); yakov.bodrov@list.ru (Y.V.B.); valeria_pavl.1@mail.ru (V.A.P.); sashai@inbox.ru (E.V.B.); zhernova.d@yandex.ru (D.A.Z.); pushkova18@gmail.com (E.N.P.); mnv-4529264@yandex.ru (N.V.M.); 2 I.M. Sechenov First Moscow State Medical University, Moscow 119991, Russia; 3 Federal Research Center for Bast Fiber Crops, Torzhok 172002, Russia; tatyana_rozhmina@mail.ru (T.A.R.); lpkudryavtseva@icloud.com (L.P.K.); 4 Moscow Institute of Physics and Technology, Moscow 141701, Russia; 5 Lomonosov Institute of Fine Chemical Technologies, MIREA—Russian Technological University, Moscow 119571, Russia; 6 Faculty of Biology, Lomonosov Moscow State University, Moscow 119234, Russia

**Keywords:** *Colletotrichum lini*, anthracnose, flax pathogen, Nanopore, T2T genome assembly, whole-genome comparison, accessory chromosomes

## Abstract

*Colletotrichum lini* is a pathogenic fungus that infects flax and causes significant yield losses. In this study, we assembled the genomes of four highly virulent *C. lini* strains using the Oxford Nanopore Technologies (ONT, R10.4.1 flow cells) and Illumina platforms. The performance of two tools developed for telomere-to-telomere (T2T) genome assembly was compared: Verkko and Hifiasm. Prior to the assembly, ONT reads were corrected using the HERRO algorithm. Verkko generated genome assemblies of high completeness but low contiguity, while Hifiasm allowed the generation of T2T assemblies. Despite significantly different genome coverage with ONT data (25–100×), four assemblies of equal contiguity were obtained: 53.6–54.7 Mb, ten core chromosomes, and two or three accessory chromosomes. A comparative analysis of different polishing tools showed that at a certain genome coverage with the corrected ONT data (≥35×), the additional polishing of the assembly did not improve its accuracy, even with the Illumina data. An analysis of the genome structures of the four *C. lini* strains revealed a high similarity between the core chromosomes. Thus, our approach enabled assembling T2T *Colletotrichum* genomes only from the ONT data obtained using R10.4.1 flow cells and may be promising for other fungal genera. These assemblies will allow the accurate identification of strain-specific differences at the chromosome level and will aid in the development of effective strategies to protect flax from anthracnose.

## 1. Introduction

Economic profits from product manufacturing depend on the availability of raw materials for production. However, yield maintenance can be a challenging task for the agribusiness sector. Cultivating plants faces a number of threats, including pathogens and unfavorable environments [1]. Flax is a multipurpose crop grown for producing seed and fiber demanded by many industries [2,3]. The species is susceptible to a number of fungal pathogens posing a threat to its yield [4]. *Colletotrichum lini* is one of the most devastating fungal pathogens causing substantial damage to flax [5,6]. The species comprises strains of different virulence, which cause flax anthracnose of varying severity [7]. The difference is determined by virulence characteristics at the genetic level [8,9]. Thus, knowledge of pathogenicity determinants at the molecular level can be employed for the effective control of the disease.

Therefore, the accuracy of the chosen strategy against the pathogen stems from the precision of the used data. Moreover, the complete picture of virulence features can be better seen at the genomic level [10,11,12]. Pathogenicity determinants can be encoded in the unique accessory chromosomes of the pathogen [13,14]. Such chromosomes can be unique to a number of strains. The alignment of genomic regions to each other or to a reference assembly can help in studying the origin and evolution of virulence characteristics [15]. However, the information on these features can be missing from the fragmented reference assemblies and impede the whole analysis. During the last decade, the number of sequenced pathogen genomes has grown incredibly fast [16]. Nonetheless, many of the genome assemblies remain at the non-chromosome level, concealing important information on the genome structure.

Third-generation sequencing technologies opened the door to high-quality genome assemblies. The Oxford Nanopore Technologies (ONT) platform produces the longest sequencing reads. The technology underwent an upgrade to new chemistry and improved sequencing accuracy and homopolymer resolution [17,18]. However, R10.4.1 flow cells allow for obtaining lower volumes of data compared to R9.4.1 flow cells. Nonetheless, the increased fidelity allows for assembling high-quality genomes [19].

Genome assemblies of the chromosome level should demonstrate the most detailed picture of the genome structure of a species representative. For pathogenic fungi, such a high level of completeness is particularly important. Pathogenicity genes can be encoded in separate chromosomes [20,21]. The upregulation of certain genes on pathogenicity chromosomes leads to impaired virulence of the fungus [15]. The horizontal transfer of entire pathogenicity chromosomes can impart pathogenicity to the non-pathogenic strains of the same species, whereas the lack of pathogenicity regions attenuates virulence in the host plant [19,22]. In addition to studying pathogenicity chromosomes, chromosome-level assemblies allow investigating the history of chromosomal rearrangements [15]. Pathogenicity genes are located in highly plastic transposon-rich genomic regions [23]. Parts of accessory chromosomes can be found in core chromosomes [24]. Thus, tracing the origin of genome regions might suggest the evolution of fungal pathogenicity [25]. The study of chromosomal structure can be helpful in both interspecies and intraspecies studies, as the transfer of the pathogenicity chromosome may confer specificity to a different host plant [26].

Complete chromosome-level assemblies allow the revealing of large variations within species representatives. In addition, unique rearrangements or genomic regions of a strain can be recognized only in de novo assemblies. Since such genomic features can be associated with pathogenicity, producing complete de novo assemblies becomes extremely important. This study aimed to construct four telomere-to-telomere (T2T) assemblies of the flax pathogen *C. lini*. In our previous works, we assembled the genomes of several *C. lini* strains [27,28,29]. However, the approach used was rather time- and resource-consuming compared to the one used in this study. In this work, we identified a rapid and effective approach to assemble a fungal genome into T2T chromosomes.

## 2. Materials and Methods

### 2.1. Fungal Material

*C. lini* strains #390-3, #391-4, and #655-1 were provided by the Institute for Flax (Torzhok, Russia) in tubes containing potato dextrose agar, 39 g/L (Condalab, Madrid, Spain). To evaluate the virulence level of the *C. lini* strains, thirteen flax varieties were used. According to the available information, two varieties were susceptible to anthracnose (Entre-Rios and Punjab), seven varieties were resistant (Crystal × C-255, C-255 × R-130-3, Leona, R-130-3, R-130-3 × Crystal, R-130-3 × (Leona × Aoyagi), and R-130-3 × R-138), and the other four varieties were moderately resistant (C-255, C-255 × A-93, Leona × Crystal, and R-138 × Crystal). For virulence level tests, ten seeds per pot were sown for each variety in three biological replicates. One-week-old seedlings were inoculated by spraying with a suspension of pathogen spores at a concentration of 150–300 spores/cm^3^. After inoculation, the plants were covered with plastic bags and incubated for 48 h. On the sixth day after inoculation, the average percentage of dead infected plants was evaluated and a virulence level was assigned to each strain: 0–30%—low virulence; 31–50%—moderate virulence; and 51–100%—high virulence.

### 2.2. DNA Extraction

For DNA extraction, we used our previously developed protocol [30] with several modifications [28]. To evaluate the quality and quantity of the extracted DNA, we used spectrophotometry (NanoDrop 2000C; Thermo Fisher Scientific, Waltham, MA, USA), fluorometry (Qubit 4.0; Thermo Fisher Scientific, Waltham, MA, USA), and agarose gel electrophoresis (2% agarose). The obtained DNA with A260/280 ~1.9, A260/230 ~2.4, and a concentration of ~400 ng/μL was used to prepare the ONT and Illumina libraries.

### 2.3. DNA Library Preparation and Sequencing on the Oxford Nanopore Technologies and Illumina Platforms

The SQK-LSK114 Ligation Sequencing Kit (ONT, Oxford, UK) was used for ONT library preparation. Sequencing was conducted on a PromethION instrument, utilizing an R10.4.1 flow cell (ONT, Oxford, UK).

The Illumina libraries were prepared using a NEBNext Ultra II DNA Library Prep Kit for Illumina (New England Biolabs, Ipswich, MA, USA) following the manufacturer’s protocol. Library quality and concentration were assessed using the Qsep1-Plus capillary electrophoresis system (Bi-Optic, New Taipei City, Taiwan) and the Qubit 4.0 fluorometer (Thermo Fisher Scientific, Waltham, MA, USA), respectively. Sequencing was performed on a NovaSeq 6000 instrument (Illumina, San Diego, CA, USA) with a read length of 150 + 150 b.

### 2.4. Genome Assembly and Polishing

Basecalling of the obtained ONT reads was performed using Guppy 6.0.1 and the configuration file dna_r10.4_e8.1_sup.cfg with min_qscore = 10 quality filtration threshold. For removing adapters, Porechop 0.2.4 (https://github.com/rrwick/Porechop, accessed on 18 October 2024) was used. The obtained Illumina reads were processed using Cutadapt 2.8 (-a AGATCGGAAGAG -A AGATCGGAAGAG) [31] and Trimmomatic 0.39 (PE, SLIDINGWINDOW:3:28, MINLEN:50) [32]. Assemblies of the *C. lini* #394-2 and #390-3 genomes were produced by Verkko 2.1 [33] and Hifiasm 0.19.9-r616 [34], and assemblies of the #391-4 and #655-1 genomes were produced by Hifiasm 0.19.9-r616. ONT reads of a length from 10 to 50 kb were separated with SeqKit v2.4.0 [35] and corrected using the HERRO algorithm [36] with Dorado 0.7.3 (correct script) (https://github.com/nanoporetech/dorado, accessed on 18 October 2024). Reads longer than 50 kb were also separated using SeqKit v2.4.0 and used without correction as ultra-long reads during the assembly. After assembling, the obtained contigs were sorted by length with SeqKit v2.4.0.

The obtained genome assemblies of strains #394-2 and #390-3 were used for testing polishing tools. The assemblies were polished with ONT reads with Medaka 1.5.0 (https://github.com/nanoporetech/medaka, accessed on 18 October 2024) and Racon 1.4.20 [37]. HyPo v1.0.3 (https://github.com/kensung-lab/HyPo, accessed on 18 October 2024), NextPolish v1.4.0 [38], Pilon 1.24 [39], POLCA (MaSuRCA 4.1.0) [40], Polypolish 0.6.0 [41], and Racon 1.4.20 were used for polishing with Illumina reads. If required, the prior alignments before polishing were produced with Minimap2 [42] and BWA 0.7.17-r1188 [43]. The obtained genome assemblies of strains #391-4 and #655-1 were polished with Illumina reads using Pilon.

To analyze the quality of the obtained assemblies, statistics of completeness, contiguity, and accuracy were calculated using BUSCO 5.3.2 (glomerellales_odb10) and QUAST 5.0.2 [44,45]. The following reference genome was used for QUAST reference-based statistics: *Colletotrichum higginsianum* IMI 349063 (NCBI Genome, GCA_001672515.1).

The mitochondrial genomes were identified in the assemblies produced by Canu 2.2 (-nanopore-raw; -minInputCoverage = 5; -stopOnLowCoverage = 5; -genomeSize = 55 m) [46] and polished with Racon 1.4.20 (ONT reads, two iterations), Medaka 1.5.0 (ONT reads), and POLCA (MaSuRCA 4.1.0; Illumina reads)—our previously optimized scheme for *C. lini* genome assembly [27]. The sequence of the previously obtained mitochondrial genome of *C. lini* strain #394-2 (NCBI GenBank, CM093684.1) [28] was blasted against the Canu-produced genome assemblies of strains #394-2, #390-3, #391-3, and #655-1 using local command line BLAST (Basic Local Alignment Search Tool) [47].

### 2.5. Genome Analysis

Tidk 0.2.31 was used for the identification and visualization of the telomeric repeat TTAGGG (https://github.com/tolkit/telomeric-identifier, accessed on 18 October 2024). The produced *C. lini* genome assemblies were aligned to the previously obtained assembly of *C. lini* strain #394-2 (NCBI Genome, GCA_043790985.1) [28] using LAST 1471 (https://gitlab.com/mcfrith/last, accessed on 18 October 2024). The Circlize [48] R package (https://www.R-project.org/, accessed on 28 November 2024) was used to visualize the comparison of genome assemblies of *C. lini* strains.

## 3. Results

### 3.1. Virulence Level of Four C. lini Strains

The virulence level of *C. lini* strains #390-3, #391-4, and #655-1 was evaluated using thirteen flax varieties. After inoculation with *C. lini* spores, on average 74.9% of flax plants died for strain #390-3, 64.5% for strain #391-4, and 56.8% for strain #655-1. The same evaluation was previously performed by us for strain #394-2 and the percentage was 69.6% [28]. Thus, all four strains were highly virulent.

### 3.2. Assembly and Polishing of Four C. lini Genomes

The genome of the highly virulent *C. lini* strain #394-2 was previously sequenced by us using the ONT and Illumina platforms [28]. ONT sequencing produced 4.1 Gb with the read N50 of 14.5 kb. Illumina sequencing produced 16 million reads of 150 + 150 b. After basecalling with the quality filtration threshold of Q10, we received 2.4 Gb with the read N50 of 14.1 kb [28]. The *C. lini* strain #394-2 genome was previously assembled by us according to the Canu—Racon × 2—Medaka—POLCA scheme and manually refined to the complete level. It had a length of 53.7 Mb and consisted of ten core and two accessory chromosomes [28]. In the present study, the sequencing data for this strain were used for testing the performance of Hifiasm and Verkko tools which became possible due to the HERRO algorithm of ONT read correction [36].

After the correction, we obtained 1.4 Gb with reads of 10–50 kb (N50 = 20.3 kb), which corresponded to ~25× genome coverage. Reads of a length of more than 50 kb (89 Mb, 1.5× genome coverage) were separated and used without correction as ultra-long reads during assembling. The assembly produced by Verkko consisted of 39 contigs, had a length of 54.2 Mb, N50 of 4.4 Mb, and BUSCO completeness of 96.7%. The assembly generated by Hifiasm consisted of 14 contigs with a length of 53.6 Mb, N50 of 5.8 Mb, and BUSCO completeness of 96.4%. Thus, the Hifiasm-produced assembly significantly outperformed the Verkko-produced assembly in terms of contiguity but was slightly inferior in terms of BUSCO completeness. The assembly parameters were also analyzed using the reference-based QUAST statistics (Supplementary Table S1) and it was suggested that the lower BUSCO completeness of the Hifiasm-generated assembly was largely because of the lower sequence accuracy compared to the Verkko-generated assembly. For example, 4462 (Hifiasm) vs. 4288 (Verkko) mismatches per 100 kbp of the reference genome (*C. higginsianum* IMI 349063, GCA_001672515.1) were revealed.

To further analyze the assembly completeness, we evaluated and visualized the frequency of telomeric repeat occurrence in the *C. lini* strain #394-2 genome assemblies. According to the obtained plot (Supplementary Figure S1), the assembly produced by Hifiasm had eight T2T chromosomes, two contigs with telomeric repeats at one end, and four contigs without any telomere. The assembly generated by Verkko had three T2T chromosomes, eight contigs with telomeric repeats at one end, and 28 contigs without any telomere (Supplementary Figure S2). This result strengthened Hifiasm’s lead.

Next, we searched for the mitochondrial genome in the produced assemblies using the previously obtained mitochondrial genome of *C. lini* strain #394-2 (CM093684.1). Both Hifiasm- and Verkko-generated assemblies were missing the mitochondrial genome. We performed the search for mitochondrial genome in the corrected and uncorrected ONT reads of a length from 10 to 50 kb. The uncorrected reads contained sequences of the mitochondrial genome, while the corrected reads had no mitochondrial genome sequences. Thus, the mitochondrial genome-related reads were lost at the read correction step.

**Figure 1:**
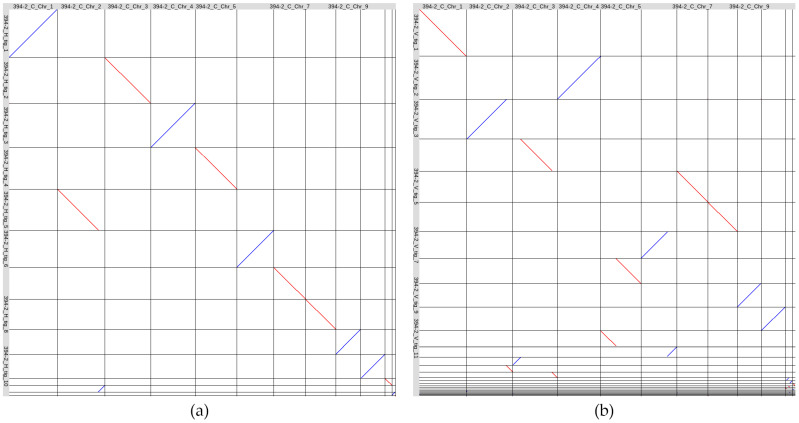
Visualization of the whole-genome LAST alignment of the *Colletotrichum lini* strain #394-2 genome assemblies (vertical axis) to the previously obtained by us complete genome assembly of this strain (GCA_043790985.1, horizontal axis): (**a**) Hifiasm-produced assembly, (**b**) Verkko-produced assembly. Red color indicates direct alignments, and blue color indicates reverse alignments. The contigs are named according to the scheme “strain number_first letter in the assembler name_contig name”.

For analyzing the structure of the obtained assemblies, we made a whole-genome alignment of the Hifiasm- and Verkko-generated assemblies of *C. lini* strain #394-2 to our previously obtained complete genome of this strain (GCA_043790985.1, [28]) (Figure 1).

According to the alignments, Verkko was not able to generate a single sequence for half of the chromosomes (Chr) (Figure 1b). Hifiasm assembled all the chromosomes except Chr 2. The end of Chr 2 was represented by a separate contig (tig_12). The other contigs were identical to those of the previously obtained complete genome of *C. lini* strain #394-2 (Figure 1a). We renamed the contigs of the Hifiasm-produced *C. lini* strain #394-2 genome assembly according to the chromosome numbers of the GCA_043790985.1 assembly: contigs 1, 6, 7, 8, 9, 10, and 11 retained their names; tig_5 became tig_2.1; tig_12–tig_2.2; tig_2–tig_3; tig_3–tig_4; tig_4–tig_5; tig_13–tig_12; tig_14–tig_13.

**Figure 2:**
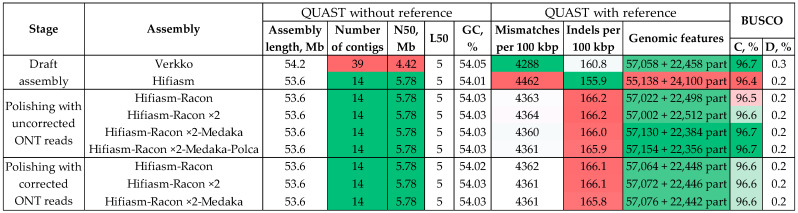
QUAST and BUSCO statistics for the *Colletotrichum lini* strain #394-2 genome assemblies produced by Verkko and Hifiasm and polished according to the Racon ×2 (ONT reads)—Medaka (ONT reads)—POLCA (Illumina reads) algorithm with the corrected or uncorrected ONT reads at each step. BUSCO: C—complete; D—duplicated. The quality of the values is indicated by the green (best)–white–red (worst) color scale. Reference genome—*Colletotrichum higginsianum* IMI 349063 (GCA_001672515.1).

Thus, the *C. lini* strain #394-2 genome assembly generated by Hifiasm was used for further polishing to improve its accuracy and completeness. We started with testing our previously developed scheme—polishing with Racon (twice, ONT reads), Medaka (ONT reads), and POLCA (Illumina reads). This algorithm proved to be the most suitable for polishing fungal genome assemblies obtained from ONT reads [27,28,29,30]. Uncorrected ONT reads were used for polishing. The QUAST and BUSCO statistics at each step are presented in Figure 2 and Supplementary Table S1. Polishing significantly decreased the number of mismatches per 100 kbp, however, the number of indels per 100 kbp rose up. Nevertheless, the number of complete reference genomic features and BUSCO completeness increased from 55,138 to 57,130 and from 96.4% to 96.7%, respectively, and became the same or better than in the Verkko-generated assembly of *C. lini* strain #394-2. To investigate the effect of read correction on polishing, we polished the Hifiasm-generated assembly using the same algorithm but with the corrected reads (Figure 2, Supplementary Table S1). There was no significant difference in the polishing results between the corrected and uncorrected reads.

**Figure 3:**
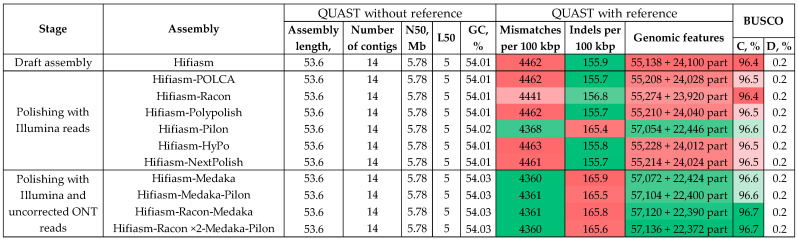
QUAST and BUSCO statistics for the *Colletotrichum lini* strain #394-2 genome assemblies produced by Hifiasm and polished with the Illumina reads and uncorrected ONT reads using different tools. BUSCO: C—complete; D—duplicated. The quality of the values is indicated by the green (best)–white–red (worst) color scale. Reference genome—*Colletotrichum higginsianum* IMI 349063 (GCA_001672515.1).

Polishing with the Illumina reads using POLCA after polishing with the ONT reads using Racon and Medaka retained the statistics almost unchanged (Figure 2, Supplementary Table S1). To test whether the stages of polishing with the ONT reads can be excluded, we applied POLCA to the draft Hifiasm-generated genome assembly of *C. lini* strain #394-2. Unexpectedly, such polishing had no effect (Figure 3, Supplementary Table S1). We decided to test more polishing tools utilizing the Illumina reads (Figure 3, Supplementary Table S1).

Pilon was the only tool that significantly improved assembly accuracy and completeness. The BUSCO completeness rose up from 96.4% to 96.6%, the number of complete reference genomic features increased from 55,138 to 57,054, and the number of mismatches per 100 kbp decreased from 4462 to 4368. Taking this fact into account, we tested different combinations of Racon, Medaka, and Pilon to find the most rapid and effective polishing algorithm for the *C. lini* strain #394-2 genome assembly (Figure 3, Supplementary Table S1). The Medaka-polished assembly gave statistics close to the Pilon-polished assembly: the number of complete reference genomic features was 57,072, the number of mismatches per 100 kbp was 4360, and the BUSCO completeness was 96.6%. The Racon—Medaka and Racon ×2—Medaka—Pilon schemes increased the BUSCO completeness to 96.7% and the number of complete reference genomic features to more than 57,120. However, there were no significant differences in mismatches and indels per 100 kbp compared to the other tested schemes (Figure 3, Supplementary Table S1). Comparing the Pilon-polished assembly with the assemblies polished according to any other used algorithm, the Pilon-polished assembly was not appreciably less accurate or less complete (Figure 2 and Figure 3, Supplementary Table S1). Considering that polishing with Pilon was performed in one step and involved precision Illumina reads unused during the assembly, we choose the Hifiasm–Pilon scheme as the final one for the *C. lini* strain #394-2 genome.

Next, we compared the produced *C. lini* strain #394-2 genome assembly with the complete genome of this strain previously obtained by us according to the Canu—Racon ×2—Medaka—POLCA scheme (GCA_043790985.1) [28]. BUSCO completeness was the same for both assemblies—96.6% (Supplementary Table S1). The number of contigs for the GCA_043790985.1 assembly was less, and the visualization of the assembly alignment (Figure 1) showed that one of the chromosomes in the Hifiasm-generated assembly was split into two contigs. The number of T2T chromosomes was eight for the assembly by Hifiasm and seven for the GCA_043790985.1 assembly. The reference-based QUAST statistics such as the number of indels and mismatches per 100 kbp and the number of complete reference genomic features were almost the same (Supplementary Table S1). Thus, both assemblies of *C. lini* strain #394-2 showed similar quality in terms of contiguity, completeness, and accuracy, but the Hifiasm–Pilon scheme was much more rapid with no need for manual contig analysis and filtering.

Three more highly virulent *C. lini* strains were sequenced on the ONT and Illumina platforms: #390-3, #391-3, and #655-1. The ONT reads were subjected to the same procedures as ONT reads obtained for the strain #394-2. The ONT data statistics are presented in Table 1. Illumina sequencing produced from 13.4 to 17.0 million reads (150 + 150 b) for each strain.

**Figure 4:**
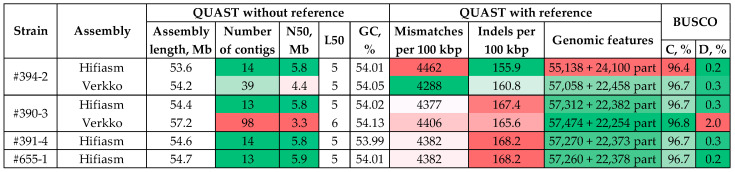
QUAST and BUSCO statistics for the genome assemblies of *Colletotrichum lini* strains #394-2, #390-3, #391-4, and #655-1 produced by Hifiasm and Verkko. BUSCO: C—complete; D—duplicated. The quality of the values is indicated by the green (best)–white–red (worst) color scale. Reference genome—*Colletotrichum higginsianum* IMI 349063 (GCA_001672515.1).

To compare the performance of Hifiasm and Verkko at a different genome coverage, we assembled the *C. lini* strain #390-3 genome, which had the highest coverage with the corrected ONT reads—100×. The QUAST and BUSCO statistics are presented in Figure 4 and Supplementary Table S2.

Both Hifiasm and Verkko produced the *C. lini* strain #390-3 genome assemblies of high BUSCO completeness—96.7% and 96.8%, respectively. However, the percentage of duplicated BUSCOs for the Verkko-generated assembly was 2%, which was unexpectedly high for the *C. lini* genome. The total assembly length of 57.2 Mb was also higher than expected (~54 Mb). The contiguity of the Verkko-produced genome assembly of strain #390-3 was even worse than that of strain #394-2 (N50 = 3.3 Mb and 98 contigs), while Hifiasm generated the strain #390-3 genome assembly of only thirteen contigs (Figure 4, Supplementary Table S2). Thus, in two cases with significantly different genome coverage (25× and 100×), the best assembly was obtained using Hifiasm.

Taking into account the obtained results, the genomes of *C. lini* strains #391-4 and #655-1 were assembled by Hifiasm. The QUAST and BUSCO statistics were high for both assemblies (Figure 4, Supplementary Table S3).

**Figure 5:**
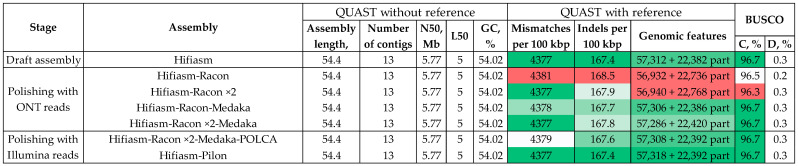
QUAST and BUSCO statistics for the *Colletotrichum lini* strain #390-3 genome assemblies produced by Hifiasm and polished with the uncorrected ONT reads and/or Illumina reads using different tools. BUSCO: C—complete; D—duplicated. The quality of the values is indicated by the green (best)–white–red (worst) color scale. Reference genome—*Colletotrichum higginsianum* IMI 349063 (GCA_001672515.1).

To test the impact of ONT data volume on the assembly accuracy, the Hifiasm-generated assembly of the *C. lini* strain #390-3 genome was polished using ONT and/or Illumina reads according to the Racon ×2—Medaka—POLCA scheme and with Pilon only, which showed high efficiency on the *C. lini* strain #394-2 genome assembly (Figure 5, Supplementary Table S2).

**Figure 6:**
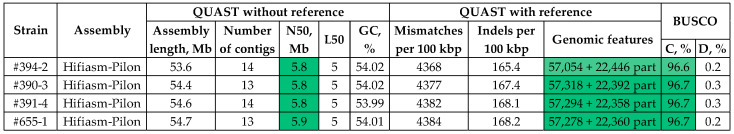
QUAST and BUSCO statistics for the *Colletotrichum lini* strains #394-2, #390-3, #391-4, and #655-1 genome assemblies produced by Hifiasm and polished with Pilon. BUSCO: C—complete; D—duplicated. The quality of the values is indicated by the green (best)–white–red (worst) color scale. Reference genome—*Colletotrichum higginsianum* IMI 349063 (GCA_001672515.1).

Pilon was the only polishing tool that made any improvement compared to the draft Hifiasm-generated assembly of the *C. lini* strain #390-3 genome. Thus, the assemblies created by Hifiasm and polished with Pilon were taken as the final for the four studied *C. lini* strains (Figure 6, Supplementary Table S3). Comparing the accuracy and completeness of the Hifiasm-produced assemblies before and after polishing with Pilon (Figure 4 and Figure 6, Supplementary Table S3), it can be seen that they were almost the same for three out of four assemblies (#390-3, #391-4, and #655-1) that is reflected in the number of mismatches and indels per 100 kbp, number of complete reference genomic features, and BUSCO completeness. We suggested that the polishing (with the ONT and/or Illumina reads) of the *C. lini* genome assembled from a certain volume of the corrected ONT R10.4.1 reads (genome coverage ~35× or higher) has a minimal effect on the assembly accuracy and completeness. All the obtained assemblies were checked for the presence of telomeric repeats. The assemblies had from five to ten T2T chromosomes (Supplementary Figure S3).

Since the ONT reads corresponding to the mitochondrial genomes were lost at the correction step, the mitochondrial genomes of the four studied *C. lini* strains were retrieved from the assemblies obtained according to the Canu—Racon ×2 (ONT uncorrected reads)—Medaka (ONT uncorrected reads)—POLCA (Illumina reads) scheme. The corresponding contigs had a length of 39.1 kb and were included in the final genome assemblies of the four *C. lini* strains.

### 3.3. Comparative Analyses of Four C. lini Genome Assemblies

**Figure 7:**
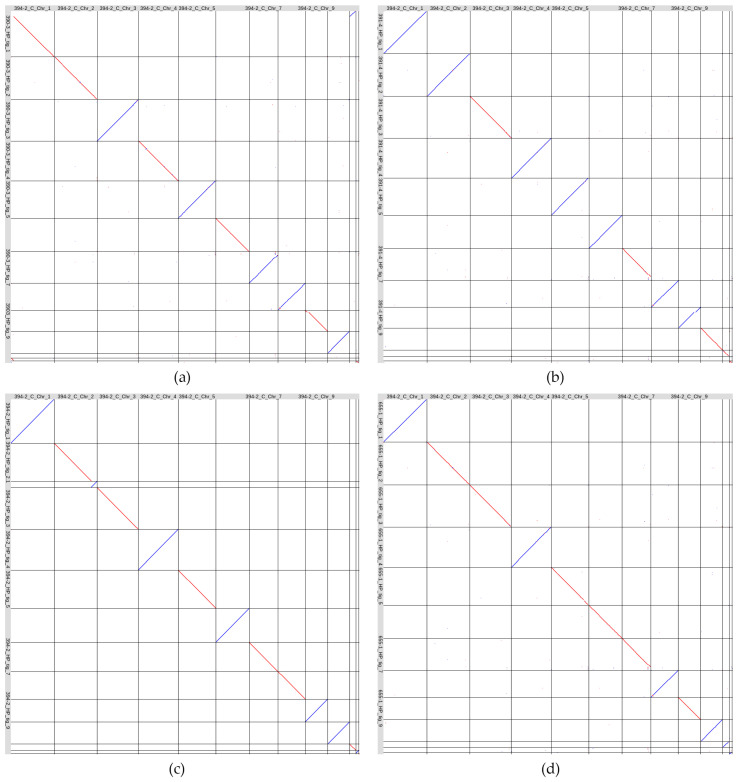
Visualization of whole-genome LAST alignments of four *Colletotrichum lini* genome assemblies produced by Hifiasm and polished with Pilon (vertical axis) to the previously obtained by us complete *C. lini* strain #394-2 genome assembly (GCA_043790985.1, horizontal axis): (**a**) strain #390-3, (**b**) strain #391-4, (**c**) strain #394-2, (**d**) strain #655-1. Red color indicates direct alignments, and blue color indicates reverse alignments. The contigs are named according to the scheme “strain number_first letter in the assembler name and first letter in the polisher name_contig name”.

To compare the obtained *C. lini* genome assemblies, we performed whole-genome LAST alignments of the strains #390-3, #391-4, #394-2, and #655-1 genome assemblies to the previously obtained by us complete genome of *C. lini* strain #394-2 (GCA_043790985.1) (Figure 7).

According to the alignments, there were no significant rearrangements between the chromosomes of any of the analyzed strains. However, tig_1 of the strain #390-3 genome assembly consisted of Chr 1 and Chr 11 of the GCA_043790985.1 assembly, and one end of Chr 1 was assembled as a separate tig_12. There were no such phenomena in the assemblies of the other tree strains, as well as in the assembly of strain #390-3 produced by Hifiasm without ultra-long reads. For strain #390-3, ultra-long reads likely led to the erroneous fusion of two different chromosomes, so we manually split tig_1 into two contigs. In addition, several small rearrangements were observed in different chromosomes of the four strains. For example, the end of Chr 7 and a region at the end of Chr 8 of the GCA_043790985.1 assembly had small rearrangements compared to the genome assemblies of strains #390-3, #391-4, and #655-1. The four *C. lini* strains had two similar accessory chromosomes, and strains #390-3, #391-4, and #655-1 had one more accessory chromosome that was not aligned to any part of the GCA_043790985.1 assembly. The smallest contigs (~0.1 Mb) in the assemblies of strains #391-4 (tig_14) and #394-2 (tig_13) represented contaminations according to BLAST analysis and were excluded from the final assemblies. Thus, all four obtained *C. lini* genome assemblies consisted of ten core chromosomes and two or three accessory chromosomes and can be considered complete genome assemblies.

**Figure 8:**
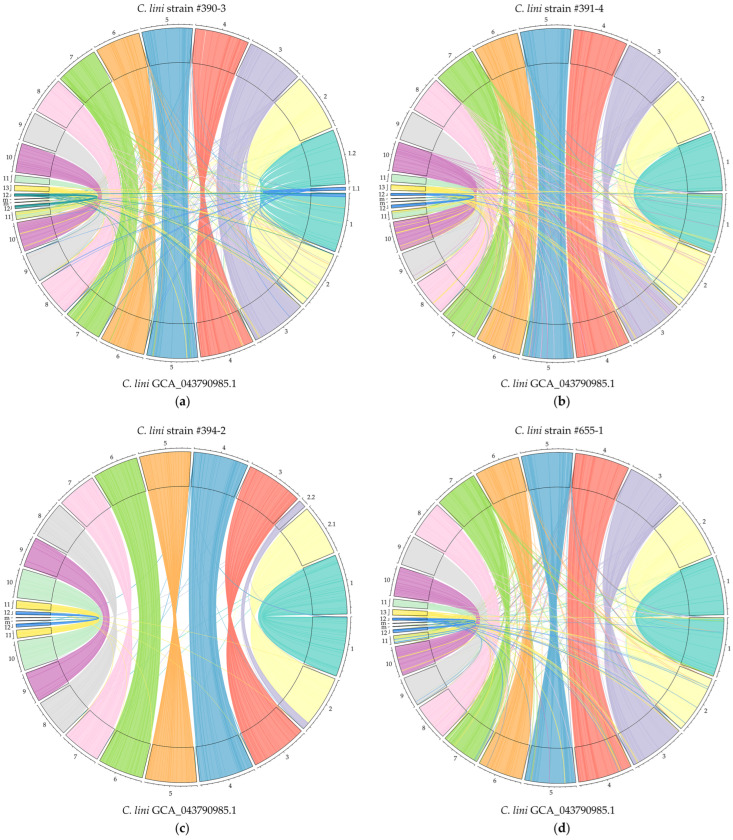
Visualization of chromosome alignment and duplication events between four *Colletotrichum lini* genome assemblies produced by Hifiasm and polished with Pilon and the previously obtained by us complete *C. lini* strain #394-2 genome assembly (GCA_043790985.1): (**a**) strain #390-3, (**b**) strain #391-4, (**c**) strain #394-2, (**d**) strain #655-1. Chromosome numbers (1–13) are shown in the outer circles, m—mitochondrial genome.

We renamed the contigs of the final *C. lini* genome assemblies according to the chromosome numbers of the GCA_043790985.1 assembly and performed the whole-genome Circlize alignments to further compare the obtained *C. lini* genome assemblies and to find the duplications between chromosomes (Figure 8). 

The analysis confirmed the high similarity between the genomes of the four *C. lini* strains and other observations on the assemblies seen in the LAST alignment (Figure 7). Figure 8c, which can be considered a self-alignment, showed that there were only a small number of self-duplications within the strain *C. lini* #394-2 genome, primarily in the telomeric regions of the chromosomes. The single chromosome that had significant duplications with other chromosomes, not only in telomeric regions, was the accessory chromosome presented in the *C. lini* strains #390-3, #391-4, and #655-1 genome assemblies (Chr 13). This accessory chromosome was similar in the assemblies of these three strains.

## 4. Discussion

Modern genomics of fungal pathogens offers vast opportunities for in-depth studies of pathogenicity at the molecular level. Complete genome assemblies became useful tools to search for pathogenicity determinants encoded in the genome and their relation to the actual virulence level. For *C. lini*, we obtained highly complete and contiguous assemblies in our previous studies [27,28]. However, the used approaches for assembling a high-quality genome had two main shortcomings. First, the used genome assembler—Canu—required a lot of computing hours to construct genome sequences. Second, the tool produced many small ambiguous contigs that needed manual curation.

This study aimed to obtain the T2T assemblies of four *C. lini* strains. Previously, hybrid strategies became popular to obtain the chromosome assemblies of different species [49,50,51,52]. Thus, the data from one platform was intended to achieve contiguity, e.g., ONT, and another one was employed to increase the accuracy, e.g., Illumina. Recently, a variety of tools were developed to produce T2T genome sequences. For instance, Verkko and Hifiasm can introduce ultra-long ONT reads when assembling high-fidelity PacBio reads [33,53]. Currently, the hybrid approach is still indispensable for the assembly of big and complex genomes [52,54,55]. However, this approach requires the use of two sequencing technologies simultaneously. Therefore, it becomes unreasonably cost- and time-consuming for small genomes. Meanwhile, the use of long-read data of a higher quality could solve the issue. Thus, read-correcting modules and tools were invented. To assemble genomes, we used two tools developed for T2T assembly (Verkko and Hifiasm) and supporting the use of ultra-long ONT reads [33,53].

We sequenced the genomes of four *C. lini* strains on the ONT R10.4.1 flow cells and obtained 2.4–10.0 Gb of basecalled data per strain. Thus, the genomes were covered 45–180 times. The sufficiency of the achieved coverage should result from both the N50 value of the sequenced reads and their mean accuracy. Since ONT reads are error-prone, the assembly from ONT data needs further correction with another type of data. Another option is to correct sequencing reads prior to genome assembly [56,57]. For instance, the Ratatosk tool was invented to correct ONT reads with precision short-read data. However, modern software offers opportunities to correct error-prone data without the use of sequencing reads from another platform. In our work, we used Dorado, which includes a module (HERRO) for single-read error correction (https://github.com/nanoporetech/dorado, accessed on 18 October 2024). Only reads longer than 50 kb were kept untouched and used as ultra-long reads during assembly. According to the HERRO recommendations, we discarded reads shorter than 10 kb. Thus, genome coverage with ONT reads decreased to a range of 25–100×. The sensitivity of an assembler to genome coverage with sequencing reads and their quality depends on the type of its assembly algorithm [29]. However, in this study, the difference in coverage values had no effect on the main QUAST statistics of contiguity (number of contigs, N50, L50) of the optimal assemblies (all produced with the same tool). Therefore, the lowest coverage (~25×) was still sufficient for producing a contiguous assembly of the fungal genome according to the chosen strategy. Nevertheless, the assemblies corresponding to 35–100× coverage had higher BUSCO completeness and higher numbers of complete reference genomic features. However, there was no correlation between these quality statistic values and the coverage values. The only significant disadvantage in using error-corrected reads resides in handling overrepresented sequences. Thus, the sequences of mitochondrial genomes were lost at the step of error correction with Dorado. Therefore, they should be obtained separately from the nuclear genome assembly, for example, by mapping uncorrected reads to the reference mitochondrial genome and further assembling the mapped reads.

To test the performance of the assembly software on the lowest coverage, we compared the performance of Verkko and Hifiasm on the genome of *C. lini* strain #394-2. The obtained assemblies had different accuracy characteristics. The assembly by Verkko had higher BUSCO completeness and number of complete reference genomic features. However, it was more fragmented than the assembly by Hifiasm. The assembly by Verkko had a higher number of contigs and a lower N50 value. In addition, according to a telomere analysis, only three contigs from the assembly by Verkko had telomeric repeats at both ends. The analysis of the telomeric repeats of the assembly by Hifiasm confirmed that the majority of the contigs had telomeric repeats at both (eight contigs) or at least one (two contigs) end. Thus, the use of ONT R10.4.1 data and the latest assembly tool Hifiasm for T2T assembly allows for restoring the sequences of whole fungal chromosomes.

The alignment of the assembly by Hifiasm to the previously obtained complete assembly of strain #394-2 (Canu—Racon ×2—Medaka—POLCA) confirmed that the two assemblies were almost identical in terms of chromosome structure. Only Chr 2 was split at one of its ends. This might be due to the complex structure of the end regions but needs further investigation. Thus, we concluded that Hifiasm obtained an assembly of a T2T level. The newer assembly strategy is advantageous to the older one in its simplicity. We decided that this assembly is most optimal at the step of the draft assembly, as the accuracy of an assembly can be further improved with polishing. Using Hifiasm, we obtained three more genome assemblies of *C. lini* strains #390-3, #391-4, and #655-1. They numbered 13–14 contigs and had N50 values of 5.8–5.9 Mb.

High nucleotide fidelity should guarantee the correctness of further genomic analysis, e.g., SNP calling, gene search, transcriptomic analysis, etc. In our previous studies, we observed the improving quality of an assembly (decrease in the relative number of indels/mismatches and increase in the number of complete BUSCOs and reference genomic features) upon polishing with ONT R9.4.1 data [27,28]. However, in this work, polishing with the ONT R10.4.1 data had a positive effect only on the assembly of *C. lini* strain #394-2, which was generated from the lowest ONT data volume, corresponding to ~25× genome coverage (indicated by a decrease in the relative number of mismatches and an increase in BUSCO completeness and the number of complete reference genomic features). For the other three strains, for which 35–100× genome coverage with ONT reads was obtained, the QUAST and BUSCO statistics reflecting assembly completeness and accuracy were the same or even better than in the ONT-polished assembly of strain #394-2. Further polishing with uncorrected or corrected ONT reads had no positive effect on the assemblies of strains #390-3, #391-4, and #655-1. Therefore, the assemblies by Hifiasm had nearly the highest possible quality. However, we assumed that the use of precision sequencing data from another platform might have a stronger positive impact on assembly quality.

Following the positive effect observed in our previous studies [27,28], we polished the assembly of *C. lini* strain #394-2 with the Illumina data (~90× genome coverage) using the POLCA tool. However, this brought only an insignificant improvement in the reference-based QUAST and BUSCO statistics. POLCA aligns the Illumina reads to an assembly, calls possible variants, and identifies and corrects errors in these variants [40]. As bioinformatics software has different algorithms with varying implementations, we tested a range of other polishers: Racon, Polypolish, Pilon, HyPo, and NextPolish. Among them, only Pilon showed a significant improvement in accuracy characteristics (relative number of mismatches, number of complete reference genomic features, and BUSCO completeness). Our previous study demonstrated that polishing with Illumina data after polishing with ONT R9.4.1 data was significantly more effective than polishing only with ONT or Illumina data [58]. However, in this work, consecutive polishing with the ONT R10.4.1 and Illumina data (with Pilon) brought a slight improvement to the strain #394-2 assembly quality. The assemblies polished with both the ONT and Illumina data and only with the Illumina data (with Pilon) had quite similar quality characteristics. Thus, we chose the strategy of assembling by Hifiasm and polishing with Pilon as the most optimal. Polishing the genome assemblies of the other three strains with the Illumina data using Pilon provided insignificant improvement in the QUAST and BUSCO statistics. Thus, at a certain level of genome coverage of the corrected ONT R10.4.1 reads (average quality ≥ Q10, length ≥ 10 kb), the accuracy and completeness of the Hifiasm-generated assemblies are already very high and cannot be improved by polishing, indicating that there is no need to obtain sequencing data from another platform, e.g., Illumina. According to our data for the genomes of *C. lini* strains, this level is about 35×.

The four assembled *C. lini* genomes had a very similar chromosome structure. In addition, all of the analyzed assemblies belonged to the strains of high virulence and included accessory chromosomes: two for strain #394-2 and three for strains #390-3, #391-4, and #655-1. Meanwhile, in our previous studies, we observed the absence of accessory chromosomes in the genome of a moderately virulent strain compared with that of a highly or lowly virulent strain [27]. In light of the results of the current study, this might be associated with different virulence. However, further confirmation should be received due to the lower quality of the assemblies from the previous works.

Recently, much attention has been drawn to the studies of *Colletotrichum* accessory chromosomes. *Colletotrichum* species can possess more than one accessory chromosome, e.g., from two to eight [15,59]. The difference in the number of accessory chromosomes was observed in strains of varying virulence. Karyotype studies showed that the isolates of *Colletotrichum kahawae* of varying virulence had different numbers of accessory chromosomes [60]. The isolates of the same level of aggressiveness possessed the same number of accessory chromosomes. Accessory chromosomes have a high content of repetitive DNA. The coding sequences of these chromosomes encode genes indispensable for fungal pathogenicity, as well as the genes uninvolved in infection [59]. In our study, we identified that the analyzed *C. lini* strains possess from two to three accessory chromosomes. Their content can be further investigated, and the role of the determined genes should be identified. However, the studies of other *Colletotrichum* species and our previous work demonstrated that accessory chromosomes encode many genes of unknown functions. Therefore, a detailed analysis of these sequences becomes a more complex task.

In this study, we obtained four T2T genomes of highly virulent *C. lini* strains using the ONT R10.4.1 sequencing data, the HERRO correction tool, and the Hifiasm assembler. The latest technological advances are designed to reduce the costs of assembling a genome. The use of a single sequencing platform and reduced requirements for computing resources make high-quality genome assemblies even more available. A set of contiguous and accurate genomes of a pathogenic species has the potential to become powerful tools for both basic and applied studies. The careful selection of strains for sequencing can gain useful information on the genetic pathogenicity determinants of the fungus. Such instruments should attract the attention of specialists developing effective strategies against plant pathogens. Therefore, T2T assemblies should become a new standard in fungal genomics.

## Figures and Tables

**Table 1:** The ONT data statistics at each step of read preparation for *C. lini* strains #394-2, #390-3, #391-4, and #655-1.

Strain	Basecalled Data Volume, Gb	Basecalled Data N50, kb	Coverage with Basecalled Data	Corrected Data Volume, Gb	Coverage with Corrected Data	Ultra-Long Read (>50 kb) Data Volume, Gb	Coverage with Ultra-Long Reads
#394-2	2.4	14.1	45×	1.4	25×	0.09	1.5×
#390-3	10.0	13.0	180×	5.4	100×	0.14	2.5×
#391-4	4.0	14.0	75×	2.0	35×	0.15	2.5×
#655-1	6.9	16.3	125×	4.1	75×	0.29	5.0×

## Data Availability

The generated dataset for this study can be found in the NCBI database under the BioProject accession number PRJNA929545.

## Supplementary Materials

The following supporting information can be downloaded at https://www.mdpi.com/article/10.3390/jof10120874/s1, Figure S1: Telomeric repeat (TTAGGG) content along the contig sequences of the Hifiasm-produced genome assembly of *Colletotrichum lini* strain #394-2; Figure S2: Telomeric repeat (TTAGGG) content along the contig sequences with length more than 2 Mb of the Verkko-produced genome assembly of *Colletotrichum lini* strain #394-2; Figure S3: Telomeric repeat (TTAGGG) content along the contig sequences of the genome assemblies produced by Hifiasm and polished with Pilon of *Colletotrichum lini* strains (a) #390-3, (b) #391-4, (c) #394-2, and (d) #655-1; Table S1: QUAST and BUSCO statistics of *Colletotrichum lini* strain #394-2 draft and polished genome assemblies; Table S2: QUAST and BUSCO statistics of *Colletotrichum lini* strain #390-3 draft and polished genome assemblies; Table S3: QUAST and BUSCO statistics of *Colletotrichum lini* strains #394-2, #390-3, #391-4, and #655-1 draft and final genome assemblies.
