# Supplementary figures and images for: Nanopore Data-Driven T2T Genome Assemblies of Colletotrichum lini Strains
Source: J Fungi (Basel). 2024 Dec 16;10(12):874. doi: 10.3390/jof10120874 (PMC11679667; doi:10.3390/jof10120874)

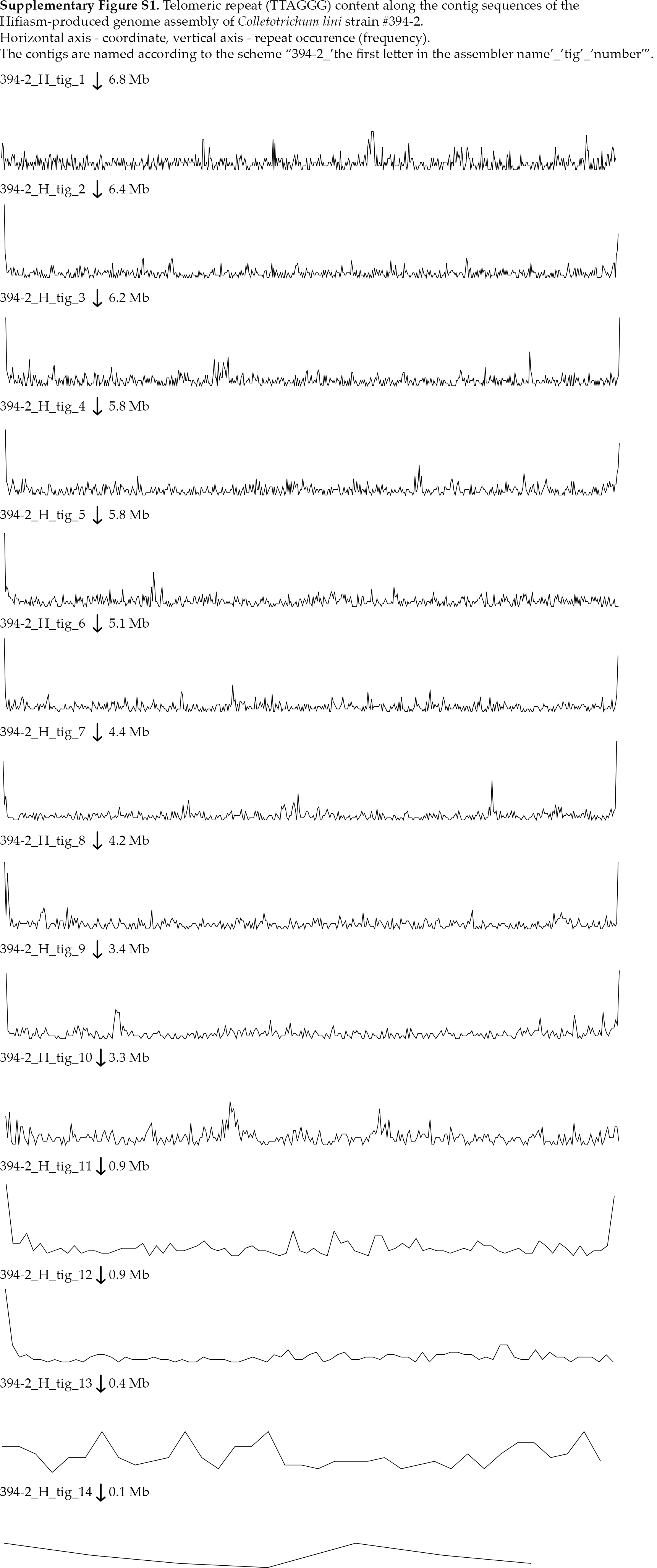

Supplement: Supplementary file 1 [file jof-10-00874-s001.zip › Figure_S1_2024-12-09.png]

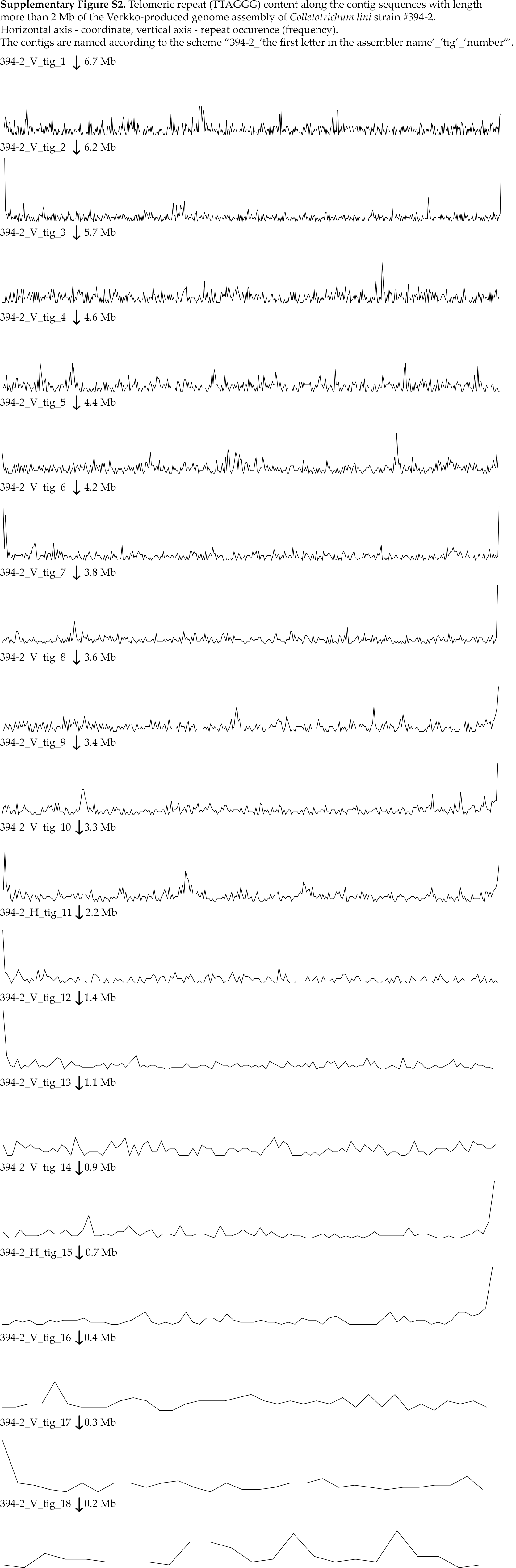

Supplement: Supplementary file 1 [file jof-10-00874-s001.zip › Figure_S2_2024-12-09.png]

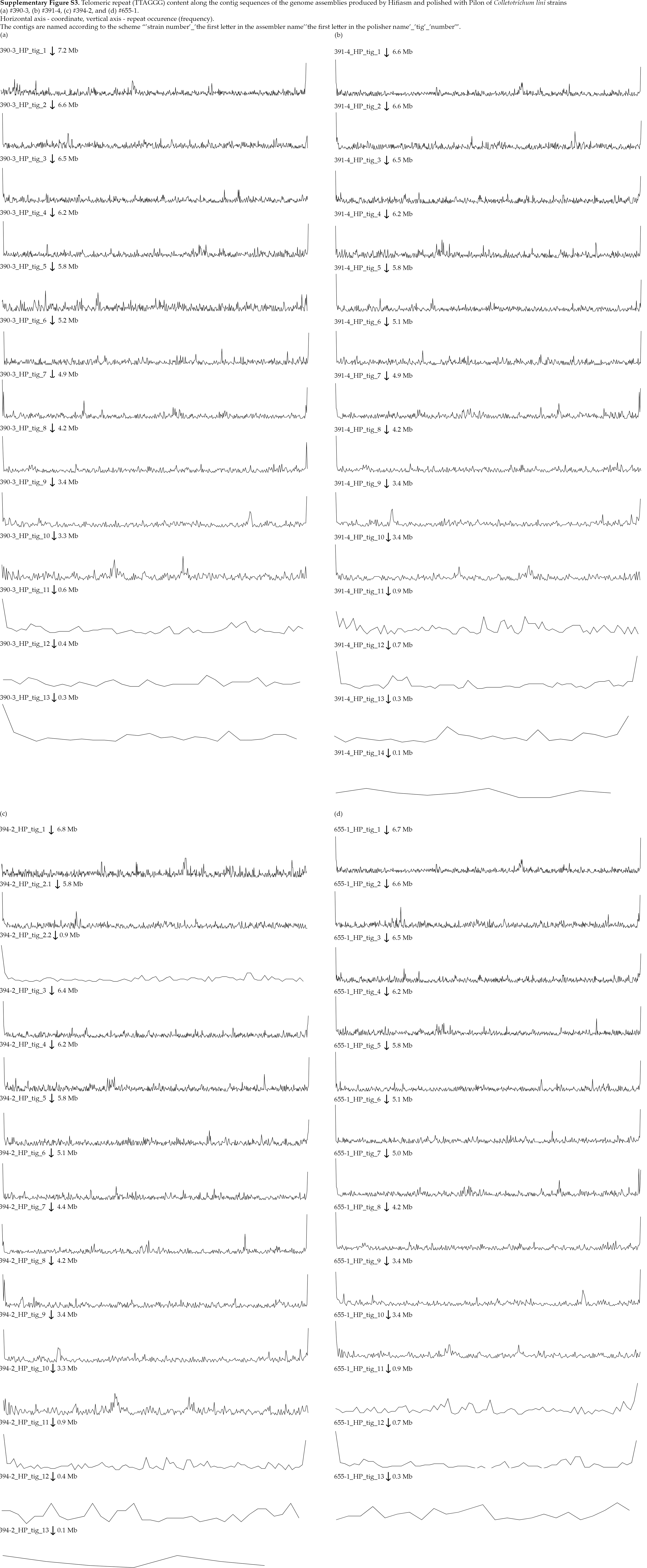

Supplement: Supplementary file 1 [file jof-10-00874-s001.zip › Figure_S3_2024-12-15.png]
